# MicroRNA 449a can Attenuate Protective Effect of Urokinase Against Pulmonary Embolism

**DOI:** 10.3389/fphar.2022.713848

**Published:** 2022-04-28

**Authors:** Ran Zhu, Wei-yi Qi, Ting-wei Liu, Fan Liu

**Affiliations:** ^1^ Department of Critical Care Medicine, The First Hospital of China Medical University, Shenyang, China; ^2^ Department of Pulmonary and Critical Care Medicine, The First Hospital of China Medical University, Shenyang, China

**Keywords:** acute pulmonary embolism, microRNA, miR449a, urokinase, uPA

## Abstract

Acute pulmonary embolism (APE) is a disabling diseases with high incidence rate and mortality rate. Although with high specificity, D-Dimer lacks specificity to assess APE, hence additional diagnostic and prognostic biomarkers are necessary. APE is widely treated with serine protease urokinase or urokinase-type plasminogen activator (uPA), which act as a catalyst for conversion of plasminogen to plasmin to resolve blood clots. However, it is unknown the role of differential expression of microRNAs (miRNAs) in protective effect of uPA against APE. Hence, we performed miRNA profiling in a hypoxia/reoxygenation (H/R) model of bronchial epithelial BEAS-2B cells *in vitro* and a APE mice model *in vivo*. Our analysis revealed that miR-34a-5p, miR-324-5p, miR-331-3p are upregulated with H/R or APE induction, whereas miR-429, miR-491-5p, and miR-449a are downregulated. The differential expression of the miRNAs was attenuated to levels comparable to control by treatment with uPA both *in vitro* and *in vivo*. *In situ* target prediction and analysis of potential functions of the target genes showed that the enrichment of biological processes and pathways were related to cell growth, proliferation, and inflammation. Ectopic overexpression of miR-449a using a mimic completely attenuated the protective effect of uPA in the H/R model *in vitro*. These results provide a group of miRNAs that could be used as markers, and the modulation of these miRNAs might have potential therapeutic benefits in patients with APE, which need to be validated in additional studies in humans.

## Introduction

Acute pulmonary embolism (APE) is defined as the obstruction of pulmonary circulation and hemodynamic collapse caused blood clot mediated blockage of pulmonary artery (Cohen et al., 2007; Geerts et al., 2008; Lang et al., 2013). Due to paucity of symptoms and appropriate diagnosis, the epidemiological details of APE are unknown. A multicenter study in China between 1997 and 2008 estimated that the incidence rate of APE was 0.1%, and male patients were obviously more than female ones (Yang et al., 2011). With the advancement of treatment protocols, the mortality rate of APE significantly decreased from 25.1 to 8.1% between 1997 and 2008 (Yang et al., 2011). However, it remains a serious disabling diseases which requires optimal therapies.

It has been well documented that APE is associated with inflammatory response and cell death, which might be mediated by mitogen activated protein kinase (MAPK), Phosphoinositide 3-kinases/protein kinase B (PI3K/Akt), and nuclear factor—kappa beta (NF-κβ) signaling pathways (Apostolakis and Spandidos, 2013; Wang et al., 2013; Wang et al., 2014). The ischemia and pulmonary hypertension involved in APE can induce an increase in serum levels of cytokines and chemokines, including tumor necrosis factor-alpha (TNFα), interleukin (IL)-1β, IL-8, CX3CR1, CXCRL1, brain natriuretic peptide (BNP), troponin T (TnT) and D-dimer (D2D) (Zagorski et al., 2003; Wang et al., 2013; Wang et al., 2014; Zhang et al., 2017; Shi et al., 2018). These pro-inflammatory chemokines and cytokines subsequently induce infiltration of immune cells in the lungs including natural killer cells and T cells (Lang et al., 2013; Saghazadeh et al., 2015; Saghazadeh and Rezaei, 2016).

D2D is clinically used to assess APE with high sensitivity, but its specificity is poor (Contu et al., 2010; Kessler et al., 2016), so the additional biomarkers for diagnosis of APE are needed. Circulating miRNAs have been shown to be potent biomarkers for a multitude of pathophysiological conditions (Mitchell et al., 2008; van Rooij and Olson, 2009; Alevizos and Illei, 2010; Contu et al., 2010; D'Alessandra et al., 2010; Markham and Hill, 2010; Voellenkle et al., 2010; Zampetaki et al., 2010). MiR-134 and miR-1233 have been indicated as potential biomarkers for APE diagnosis (Xiao et al., 2011; Kessler et al., 2016).

Urokinase or urokinase-type plasminogen activator (uPA), a serine protease, is the most widely used drug for treating APE, which act as a catalyst for conversion of plasminogen to plasmin to resolve blood clots (Sasahara et al., 1967; Cheng et al., 2002). Indeed, dose-effect and duration-effect clinical trials have been performed to study outcome of urokinase treatment on patients with APE (Zhang et al., 2007; Wang et al., 2009; Zhao et al., 2018; Zhang et al., 2019). uPA mediates its activity by binding to its urokinase plasminogen activator receptor (uPAR) (Xu et al., 2020). Exogenous uPA has been shown to induce expression of uPAR (Toki et al., 1985). Interaction of uPA with uPAR is critical in APE resolvement (Bdeir et al., 2000; Liu et al., 2008). In breast cancer, it has been shown that miR-645 can target uPA (Meng et al., 2018). However, it is unknown the role of differential expression of miRNAs in protective effect of uPA against APE.

Hence, our study determined differential expression of miRNA in an model of APE (hypoxia/reoxygenation) with or without uPA treatment *in vitro*, which were verified in an mice model of APE *in vivo*. Moreover, *In situ* prediction algorithms were used for target prediction of differentially expressed miRNAs and GSEA.

## Materials and Methods

### Hypoxia/Reoxygenation Model *In vitro* and Treatments

Human bronchial epithelial cells (BEAS-2B) (ATCC) were cultured in DMEM containing 5% FBS, 100 U/ml penicillin, and 100 μg/ml streptomycin, and kept in incubators with 5% CO_2_ at 37°C. To establish the hypoxia/reoxygenation (H/R) model, BEAS-2B cells were exposed to hypoxic condition (1% O_2_, 5% CO_2_, and 94% N_2_) in serum and glucose free DMEM for 12 h. After the incubation under hypoxic conditions, the culture medium was replaced with normal growth medium and cells were incubate for an additional 12 h under normoxic conditions (5% CO_2_), which were used as H/R group. Then cells were treated with recombinant urokinase (United Kingdom; 10 ng/ml, American Diagnostica, Stamford, CT), which were used as H/R + United Kingdom group. Cells only cultured under normoxic conditions were used as controls, and normal cells treated with recombinant urokinase were used as United Kingdom group. Before induction of H/R, cells were transfected with 30 nM of has-miR-449a mimic or negative control mimic (MISSION microRNA mimic, HMI0576 and HMC0003, respectively; Sigma Aldrich) using Lipofectamine LTX PLUS (ThermoFisher Scientific) for 72 h.

### Apoptosis Assay

The apoptosis of cells was evaluated by the TUNEL assay kit (R&D Systems). TUNEL labeled cells were counterstained with 4′,6-diamidino-2-phenylindole (DAPI) and subsequently washed thrice with phosphate-buffered saline (PBS). The apoptosis rate was defined as number of apoptotic cells/total number of cells × 100%.

### Determination of Cytokines

At the end of the experiment, cells were centrifuged at 1,000 g for 5 min at 4°C, and the obtained supernatant was used to determine the expression of cytokines, including BNP, TNFα, CX3CL1, IL-4, and IL-10. Luminex (Millipore) was used to quantify the levels of the cytokines. The cells were prepared for Western blot to determine the expression of bcl-2, Bax, Caspase-3 using routine methodologies. The primary antibodies for bcl-2, Bax, Caspase-3, and Actin were all purchased from Cell Signaling Technology. The analysis of relative band intensity was conducted with ImageJ version 1.46 (National Institutes of Health, Bethesda, United States).

### Fluorescence-Activated Cell Sorting Analysis

Cells were incubated with anti-CD11b-APC or anti-CD206-APC (Biolegend, San Diego, CA, United States) for 30 min on ice. After washing, the cells were resuspended in wash buffer with 2% FBS and assessed with a BD FACS Canto II instrument and the software “FACS Diva” (BD Biosciences, San Jose, CA, United States). The cells expressing CD11b surface markers were defined as pro-inflammatory M1 macrophages, and cells expressing CD206 surface markers were defined as anti-inflammatory M2 macrophages.

### Establishment of an Acute Pulmonary Embolism Model

All animal studies were approved by the Instituitional Animal Use and Care Committee of The First Hospital of China Medical University. BALB/c mice were purchased from Beijing Vital River Laboratory Animal Technology Co., Ltd. (Beijing, China) and housed in pathogen-free conditions with free access to food and water. APE was established using previous protocol (Song et al., 2013). Briefly, 0.2 ml of blood samples were collected by orbital bleeding and incubated with 200U of hepatothrombin for 1 h to generate autologous thrombus. The mice were anesthetized by injection of pentobarbital sodium at 50 mg/kg, then a 7F catheter was inserted *via* the right femoral vein into the right pulmonary artery. To establish the PE model, the autologous thrombus with 0.5 ml of saline was infused into the 7F catheter. The animals in the control group received saline (*n* = 6), and the mice established the APE model were used as PE group (*n* = 6). Mice model of APE received 5,000 IU/kg urokinase (United Kingdom) (ND Pharmaceuticals Co. Ltd., Nanjing, China) in 0.5 ml of normal saline within 0.5 h of PE induction was the PE + United Kingdom group (*n* = 6), normal mice received United Kingdom was the United Kingdom group (*n* = 6). The mice with APE modeling and United Kingdom treatment were injected with miR-449a mimic alongside United Kingdom, and used as thePE + UK + miR-449a mimicgroup (*n* = 6).

### Pulmonary Arterial Pressure Measurement

Six hours after APE modeling, mice were anesthetized to measure pulmonary artery pressures. A PTE50 catheter was inserted into the pulmonary artery *via* the right ventricle while the other end was connected to the pressure transducer. Stable pulmonary arterial pressure was recorded for 3 min using the Chengdu Thai Union BL-420S-TyPTE system. Mean pulmonary arterial pressure (PAMP), pulmonary diastolic (PADP) and systolic (PASP) were calculated from the recorded wavefront.

### Blood, Tissue Collection, and Hematoxylin and Eosin Staining

Blood was collected for serum isolation, which was then used to determine expression of D-Dimer (D2D), BNP, CX3CL1, IL-4, and IL-10 using Luminex as described above. Collected pulmonary tissues were fixed in 4% paraformaldehyde. After embedding and section, 5 µm slices were processed for hematoxylin and eosin (H&E) staining using routine methodologies and then imaged using an optical microscope.

### miRNA Isolation and Real Time Quantitiative Polymerase Chain Reaction

At the end of the experiment, miRNA was extracted from cells using PureLink miRNA isolation kit (Thermo Fisher Scientific) as manufacturer’s guidelines. Relative expression of miRNAs was determined using the miScript miRNA PCR Array Human Hypoxia Signaling (MIHS-121Z) (Qiagen). To determine expression of miRNAs in the serum of mice, miRNA was isolated and TaqMan probes were used to determine expression of the indicated miRNAs. Data was analyzed using the standard 2^−ΔΔCt^ method. Expression of mmu-miR-16 was used to normalize data, and log2 fold changes were visualized using heat map.

### In Silico Prediction of miRNA Targets and Functional Predictions

Putative target genes of the top 10 up and down regulated miRNAs differentially expressed among the control, H/R and H/R + United Kingdom group were predicted using the Validated Target module of the miRWalk database (http://www.umm.uni-heidelberg.de/apps/zmf/mirwalk/) (Dweep et al., 2011). The inclusion criteria used for target gene prediction were 1) *p*-value of 0.5, 2) seed sequences in miRNAs complementary to the 3′ untranslated regions (3′-UTRs), and 3) targets recognized by all three databases, including miRTarBase (http://mirtarbase.mbc.nctu.edu.tw/php/index.php) (Hsu et al., 2011), miRDB (http://www.mirdb.org/) (Wang, 2008), and TargetScan (http://www.targetscan.org/) (Agarwal et al., 2015). Functional profiling and enrichment analysis of predicted miRNA target genes were performed using g:Profiler (Reimand et al., 2007; Raudvere et al., 2019) and Enrichr (Kuleshov et al., 2016). Gene ontology (GO) and pathway analysis were performed with a false discovery rate (FDR) < 0.05 and *p* < 0.05 defined as the threshold of significance.

### Statistical Analysis

Data was expressed as mean ± standard deviation (SD). Statistical significance was determined using one-way analysis of variance (ANOVA) with statistical significance defined as *p* < 0.05.

## Results

### Treatment With Urokinase-Type Plasminogen Activator Reverses Differential miRNA Expression Following Induction of Hypoxia/Reoxygenation

**FIGURE 1:**
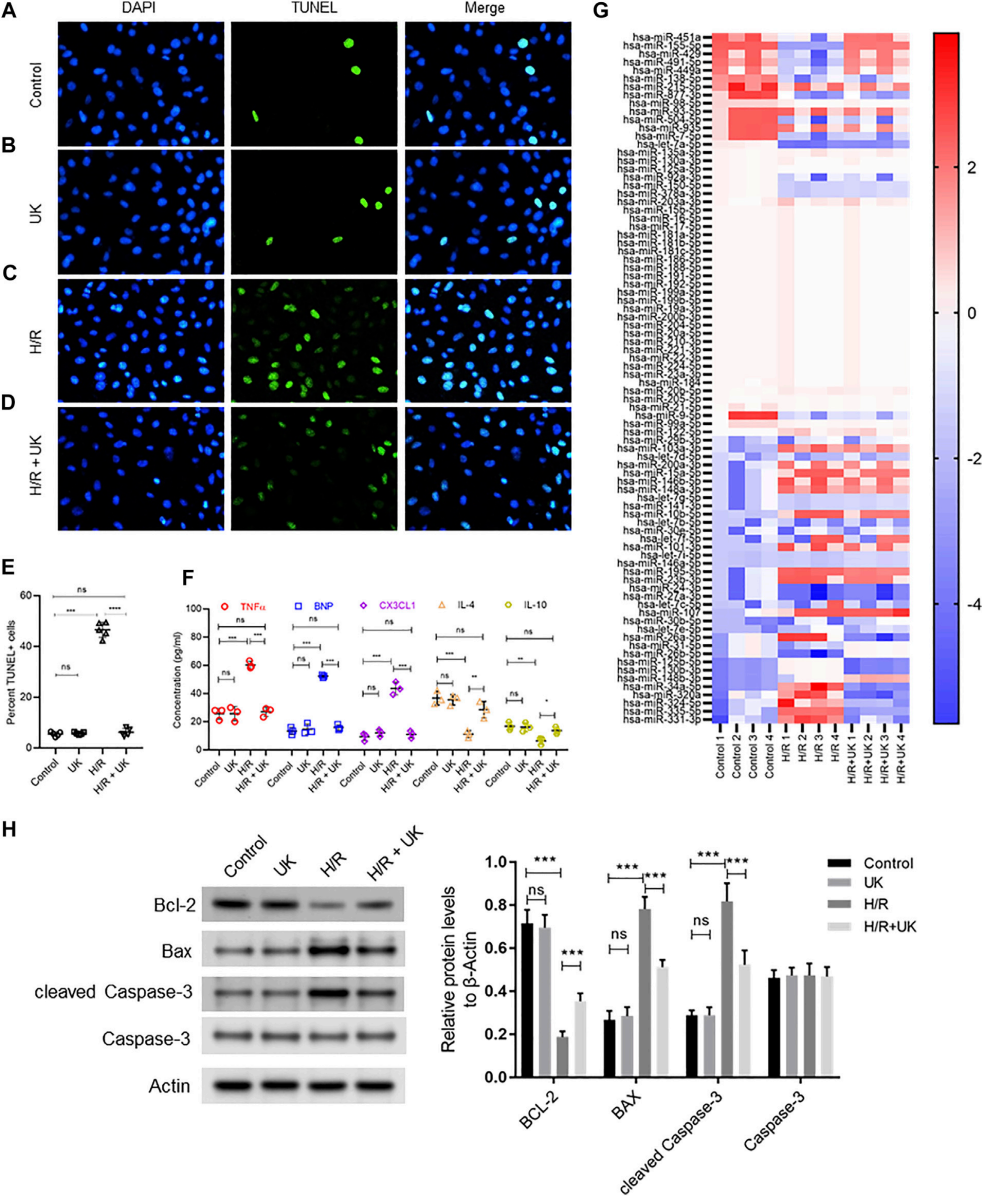
Apoptosis, inflammatory response and differential expressed miRNAs in hypoxia/reoxygenation (H/R) model *in vitro*. An APE model in BEAS-2B cells *in vitro* was established by hypoxic conditions for 12 h and reoxygenation for 12 h (for details refer to *Materials and Methods*). **(A–D)** Urokinase inhibits H/R induced cell death. Representative images from TUNEL assay in BEAS-2B cells grown under control conditions **(A)** or treated with uPA **(B)** or subjected to H/R **(C)** or subjected to the combination of H/R and uPA **(D)**. **(E)** Quantification of images in ***A-D***. ****p < 0.0001, *ns*, not significant (*n* = 5). **(F)** Urokinase inhibits H/R induced inflammatory response. Secretion of indicated cytokines in the cell supernatant was determined using Luminex assay. ****p < 0.0001, *ns*, not significant (*n* = 5). **(G)** Heat map showing log2 fold changes of miRNA expression in BEAS-2B cells maintained under control conditions or subjected to HR or subjected to the combination of H/R and uPA (United Kingdom) (*n* = 4). **(H)** Urokinase inhibits H/R induced apoptosis. Western blot images (left) and their quantified results (right) in BEAS-2B cells with different treatments.

The cell death and expression of apoptosis-related proteins, bcl-2, Bax and Caspase-3, in the normal cells with uPA treatment showed no significant difference with the control (Figures 1A,B,E,H). Compared to control cells, H/R significantly induced cell death assessed by TUNEL staining, and increased expression of Bax and cleaved Caspase-3, but decreased bcl-2 expression by Western blot (Figures 1A,C,E,H). Treatment with uPA significantly decreased apoptosis and expression of Bax and cleaved Caspase-3, but increased bcl-2 expression in cells with H/R induction to the level comparable to that in control cells (Figures 1C–E,H). Compared to control cells, H/R significantly increased expression of BNP, TNFα, and CX3CL1, but decreased expression of IL-4 and IL-10, and treatment with United Kingdom significantly reversed the secretion of these cytokines in cell supernatants to levels comparable to that in control cells (Figure 1F). These results confirmed that the H/R model in BEAS-2B can be implemented as a system to define mechanism of action of exogenous uPA.

The top 10 up regulated miRNAs following H/R induction, whose expression significantly decreased with uPA treatment, were hsa-miR-31-5p, hsa-miR-26b-5p, hsa-miR-125b-5p, hsa-miR-130b-3p, hsa-miR-148b-3p, hsa-miR-34a-5p, hsa-miR-320a, hsa-miR-324-5p, hsa-miR-335-5p, and hsa-miR-331-3p. Conversely, the top 10 down regulated miRNAs following H/R induction, whose expression significantly increased with uPA treatment, were hsa-miR-451a, hsa-miR-155-5p, hsa-miR-429, hsa-miR-491-5p, hsa-miR-449a, hsa-miR-138-5p, hsa-miR-215-5p, hsa-miR-877-3p, hsa-miR-98-5p, hsa-miR-93-5p (Figure 1G).

### Treatment With Urokinase-Type Plasminogen Activator Reverses Acute Pulmonary Embolism-Induced Changes of miRNA Expression in an Mice Model of Acute Pulmonary Embolism *In vivo*

**FIGURE 2:**
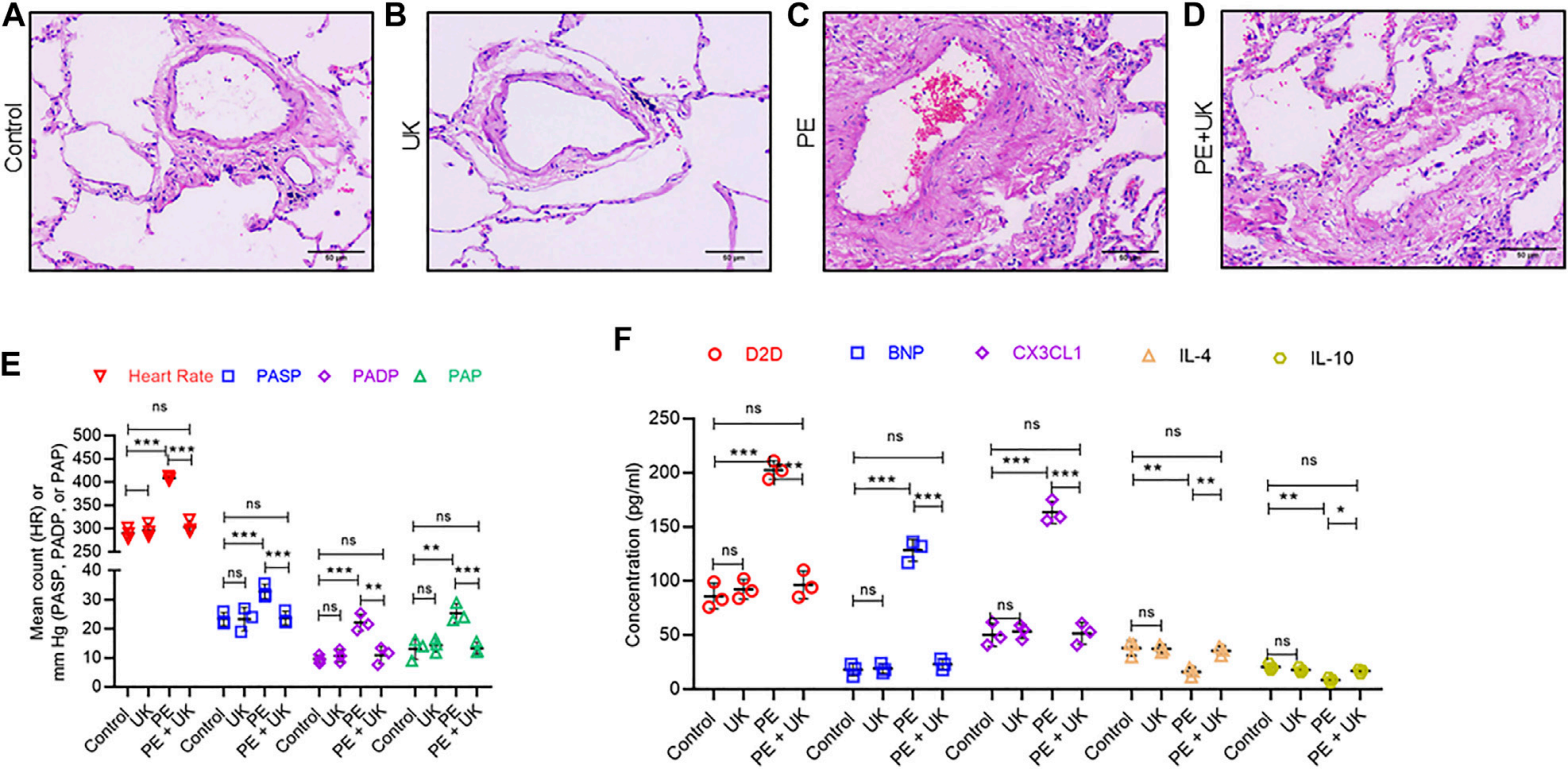
Pathological and physiological changes, and inflammatory response in an mice model of acute pulmonary embolism (APE) *in vivo*. **(A–D)** uPA inhibits H/R induced pathologicalchanges. Representative images from hematoxylin and eosin staining of bronchial tissues from mice without thrombus injection **(A)** or injected with uPA **(B)** or injected with thrombus **(C)** or injected with thrombus and uPA **(D)**. Scale bar, 100 μM. **(E)** Detection of heart rate (HR) and pulmonary artery pressure (PAP). *PASP*, pulmonary arterial systolic pressure; *PADP*, pulmonary artery diastolic pressure. Data is represented as mean ± SD; *p < 0.05, **p < 0.01, ***p < 0.001, *ns*, not significant (*n* = 6). **(F)** Serum concentration of D-Dimer (D2D), brain natriuretic peptide (BNP), Fractalkine or chemokine (C-X3-C motif) ligand 1 (CX3CL1), interleukin-4 (IL-4) and interleukin-10 (IL-10) were determined using Luminex assay. The PE group had a significant elevation of cytokines compared to the control; however, uPA treatment attenuated the increase. ****p < 0.0001, *ns*, not significant (*n* = 6).

H&E staining revealed similar lung structure in the control (Figure 2A) and mice with United Kingdom treatment (Figure 2B), and widespread lung injury in mice model of APE (Figure 2C) compared to control (Figure 2A). In mice model of APE, mixed and coagulated thrombi, perivascular edema and pulmonary abscess were visible in the pulmonary artery (Figure 2B). No thromboembolism was observed in mice in the control or United Kingdom group (Figure 2A). Treatment with urokinase resolve thromboembolism in these mice (Figure 2D). The heart rate (HR), PASP, PADP, and PAP were similar in the control and United Kingdom group, and these in the mice model of APE were significantly elevated compared with control (Figure 2E). Treatment with uPA significantly decreased HR, PASP, PADP, and PAP in mice model of APE to levels comparable to the control group (Figure 2D). Furthermore, Luminex assay found that the serum concentrations of D2D, BNP, CX3CL1, IL-4 and IL-10 were similar in the control and United Kingdom group, and concentrations of D2D, BNP, CX3CL1 in the mice model of APE were significantly increased, but concentrations of IL-4 and IL-10 were significantly decreased, which could be reversed following urokinase treatment (Figure 2F). Taken together, these results established that APE could induce thromboembolism and level changes of serum inflammatory cytokines in the mice model *in vivo*, which could be restored by treatment with uPA.

**FIGURE 3:**
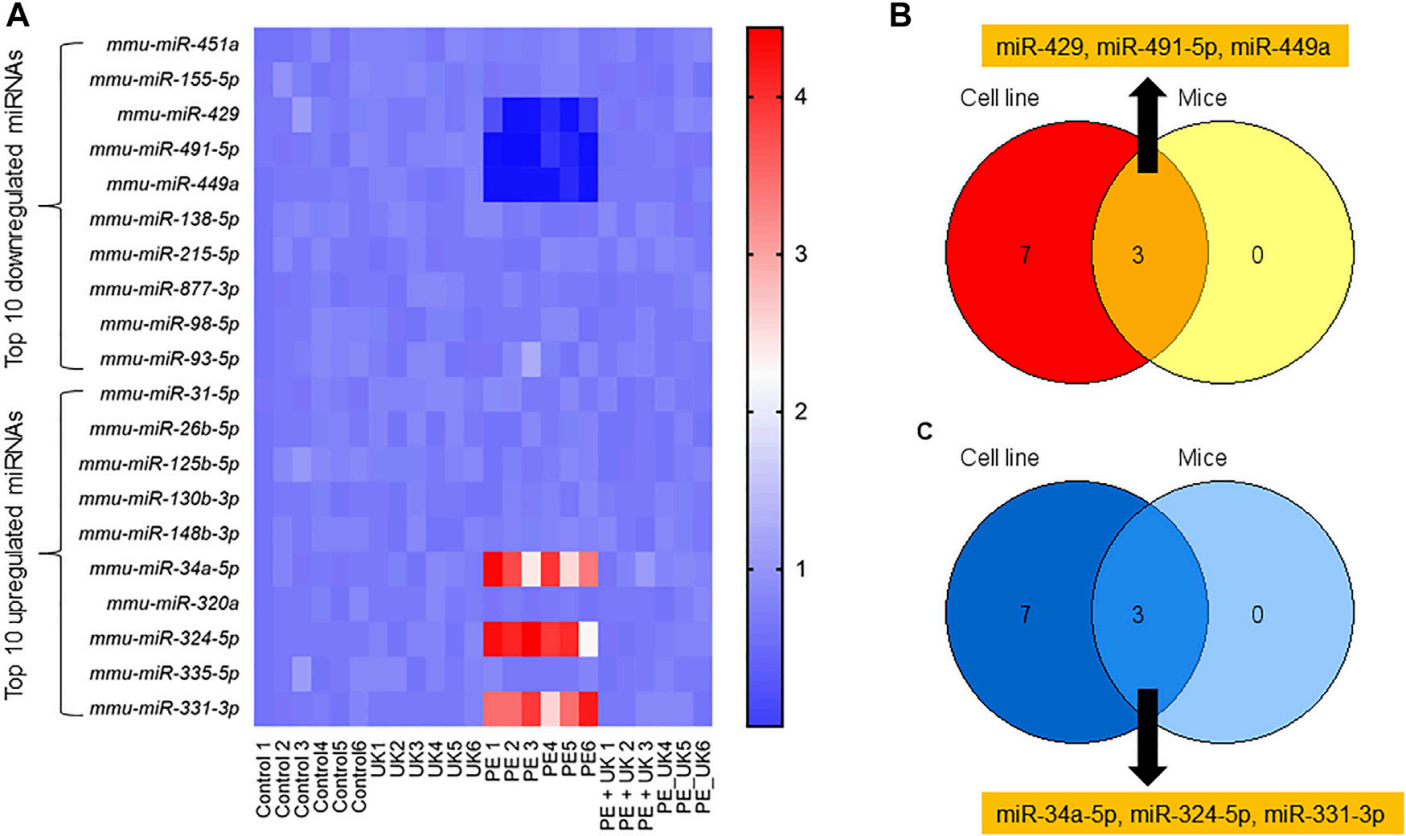
Differential expression of miRNAs post-induction of APE in mice. **(A)** Heat map of log2 fold changes of indicated miRNAs expression in mice (sham control) and subjected to APE or APE ± uPA (United Kingdom) (*n* = 6). Only top 10 up and 10 down regulated miRNAs observed in H/R model *in vitro* (**Figure1G**) were assayed. **(B,C)** Venn diagram of miRNAs down **(B)** and up **(C)** regulated in both *in vitro* and *in vivo*.

The expressions of the top 10 up and down regulated miRNAs observed *in vitro* were investigated *in vivo*. Of the top 10 up regulated miRNAs, only 3 - mmu-miR-34a-5p, mmu-miR-324-5p, and mmu-miR-331-3p *in vivo* showed similar expression pattern observed *in vitro* (Figures 3A,B). Expression of mmu-miR-34a-5p, mmu-miR-324-5p, and mmu-miR-331-3p significantly increased after induction of PE but were significantly down regulated following u-PA treatment. Similarly, of the top 10 down regulated miRNAs, only mmu-miR-429, mmu-miR-491-5p, and mmu-miR-449a *in vivo* showed similar expression pattern observed *in vitro* (Figures 3A,C). Expression of mmu-miR-429, mmu-miR-491-5p, and mmu-miR-449a significantly decreased after induction of PE but were significantly increased to levels comparable in control mice following u-PA treatment. Taken together, these results suggested that miR-34a-5p, miR-324-5p, miR-331-3p, miR-429, miR-491-5p, and miR-449a were miRNAs that were differentially expressed following PE induction *in vitro* and *in vivo*, which could be restored to levels comparable to control condition with uPA treatment.

### Target Genes and Pathways Involved With Cell Growth and Survival Are Predicted From Differentially Expressed miRNAs

To identify putative target genes of the differentially expressed miRNAs, the *in situ* Validated Target module of miRWalk version 2.0 was applied. 52 targets were obtained from the 3 upregulated miRNAs, miR-324-5p, miR-331-3p, and miR-34a-5p. After deduplication, 24 unique targets were screened (Supplementary Table S1), 20 for miR-34a-5p (*PEG10*, *MTMR9*, *TMEM109*, *MAP2K1*, *PODXL*, *PP1R11*, *FOXP1*, *BCL2*, *TGF2*, *CLOCK*, *ZDHHC16*, *CDK6*, *MDM4*, *SURF4*, *AXL*, *FUT8*, *FAM46A*, *DLL1*, *FOSL1*, and *SNTB2*) (Supplementary Figure S1A), 2 for miR-324-5p (*RAN* and *KLF7*) (Supplementary Figure S1B, S2) for miR-331-3p (*KDELR1* and *NACC1*) (Supplementary Figure S1C). Similarly, for the 3 down regulated miRNAs, miR-429, miR-449a, and miR-491-5p, 36 putative targets were predicted and 18 unique targets were screened (Supplementary Table S2), 12 for miR-449a (*E2F3*, *CDK6*, *MDM4*, *POU2F1*, *MYCN*, *CCNE2*, *ADAM10*, *LDHA*, *VPS37B*, *RDH11*, *HDAC1*, and *LEF1*) (Supplementary Figure S2A), and 3 for miR-429 (*N4BP2*, *ERRF11*, and *DLC1*) (Supplementary Figure S2B) and 3 for miR-491-5p (*SHSA6*, *RDM4B*, and *IGF2BP1*) (Supplementary Figure S2C).

**FIGURE 4:**
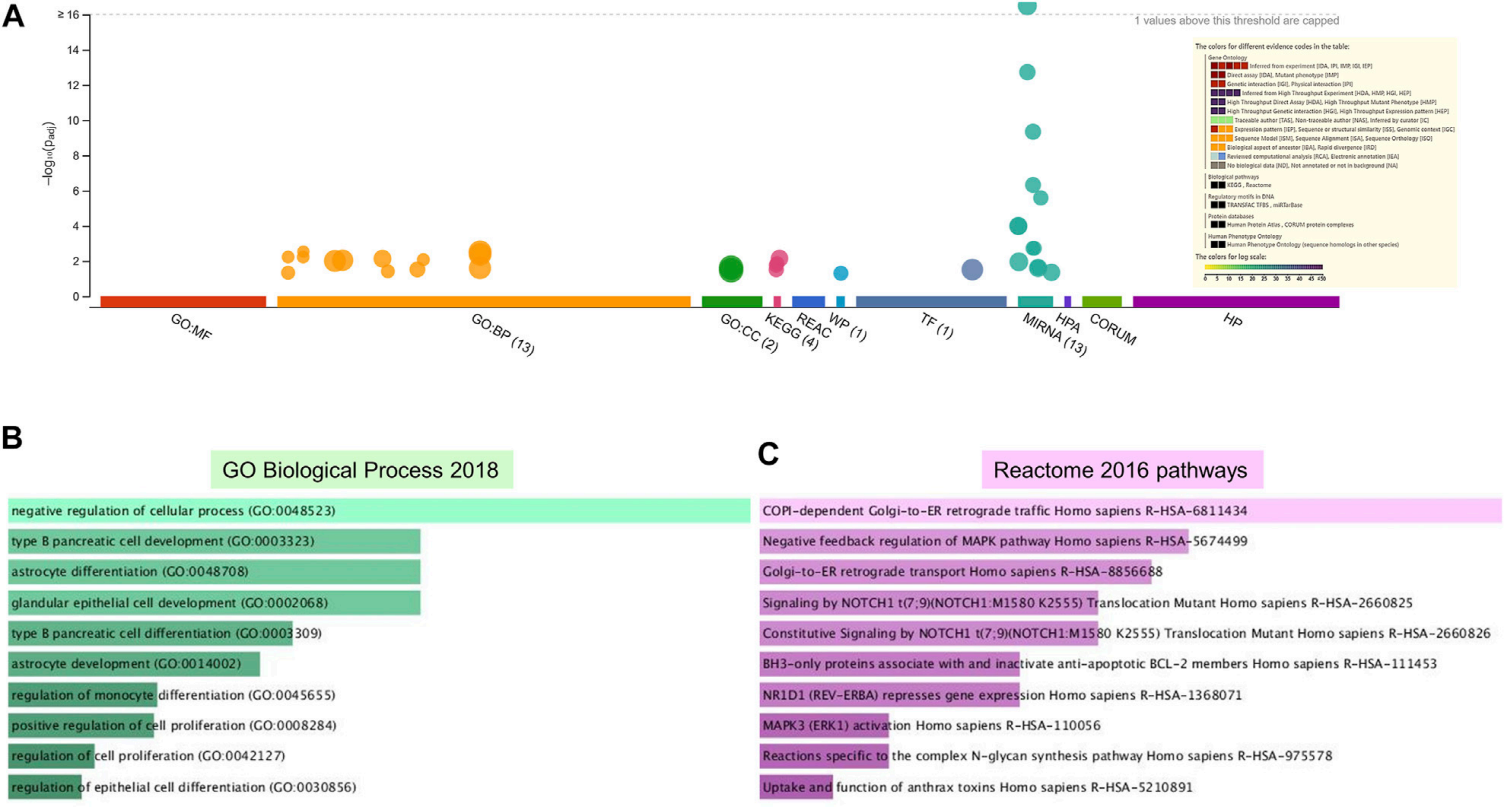
Enrichment analysis of putative targets of miRNAs up regulated in PE. **(A)** Footprint of GSEA analysis performed by g:Profiler of 24 targets. Inset shows key results. **(B,C)** Enrichment in GO biological processes **(B)** and Reaction pathways **(C)** were performed using Enrichr, with an adjusted *p* < 0.05. The length of horizontal bars indicates the number of genes in that category.

**FIGURE 5:**
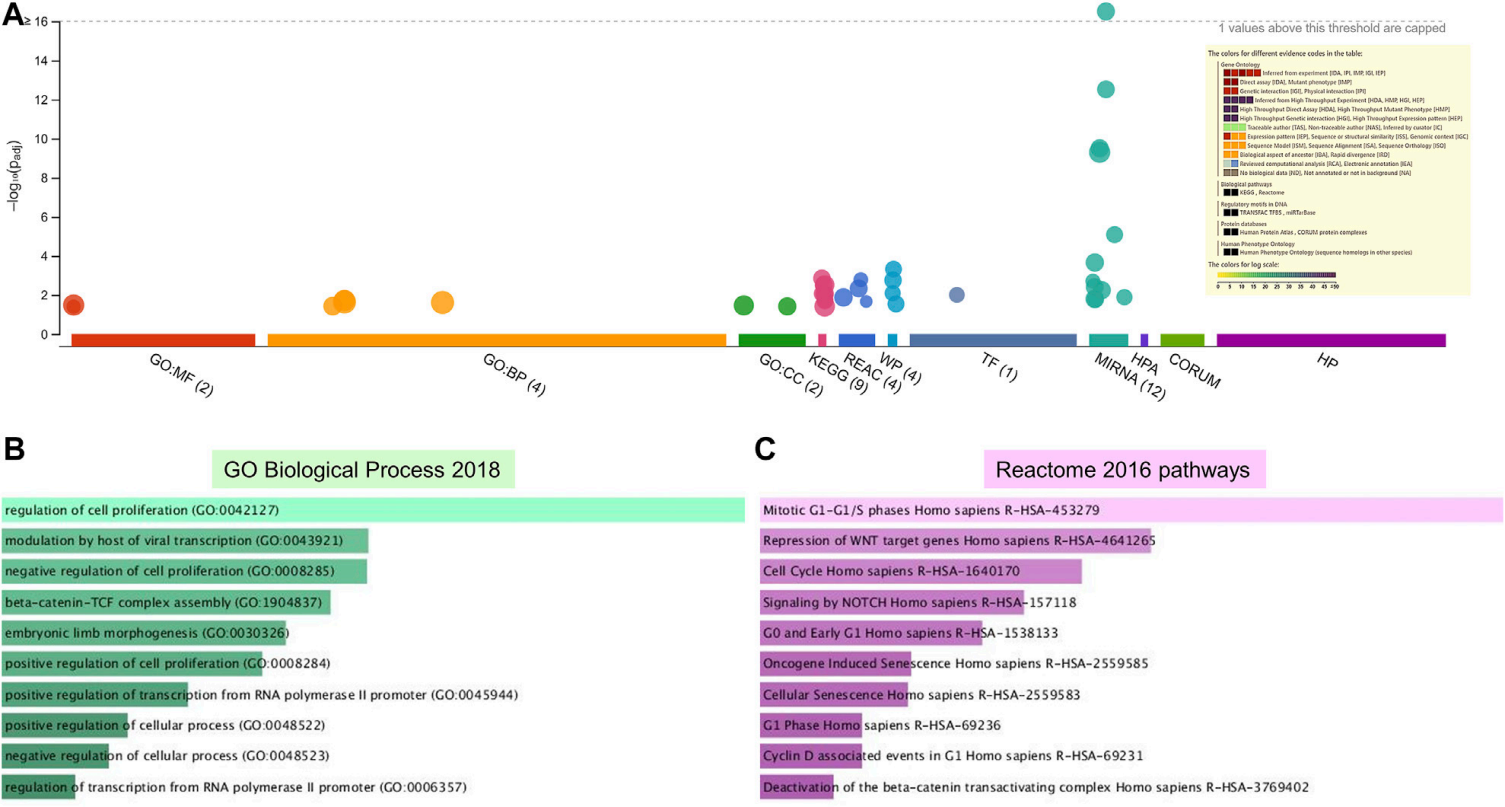
Enrichment analysis of putative targets of miRNAs down regulated in PE. **(A)** Footprint of GSEA analysis performed by g:Profiler of 18 targets. Inset shows key results. **(B,C)** Enrichment in GO biological processes **(B)** and Reaction pathways **(C)** were performed using Enrichr, with an adjusted *p* < 0.05. The length of the horizontal bars indicates the number of genes in that category.

**FIGURE 6:**
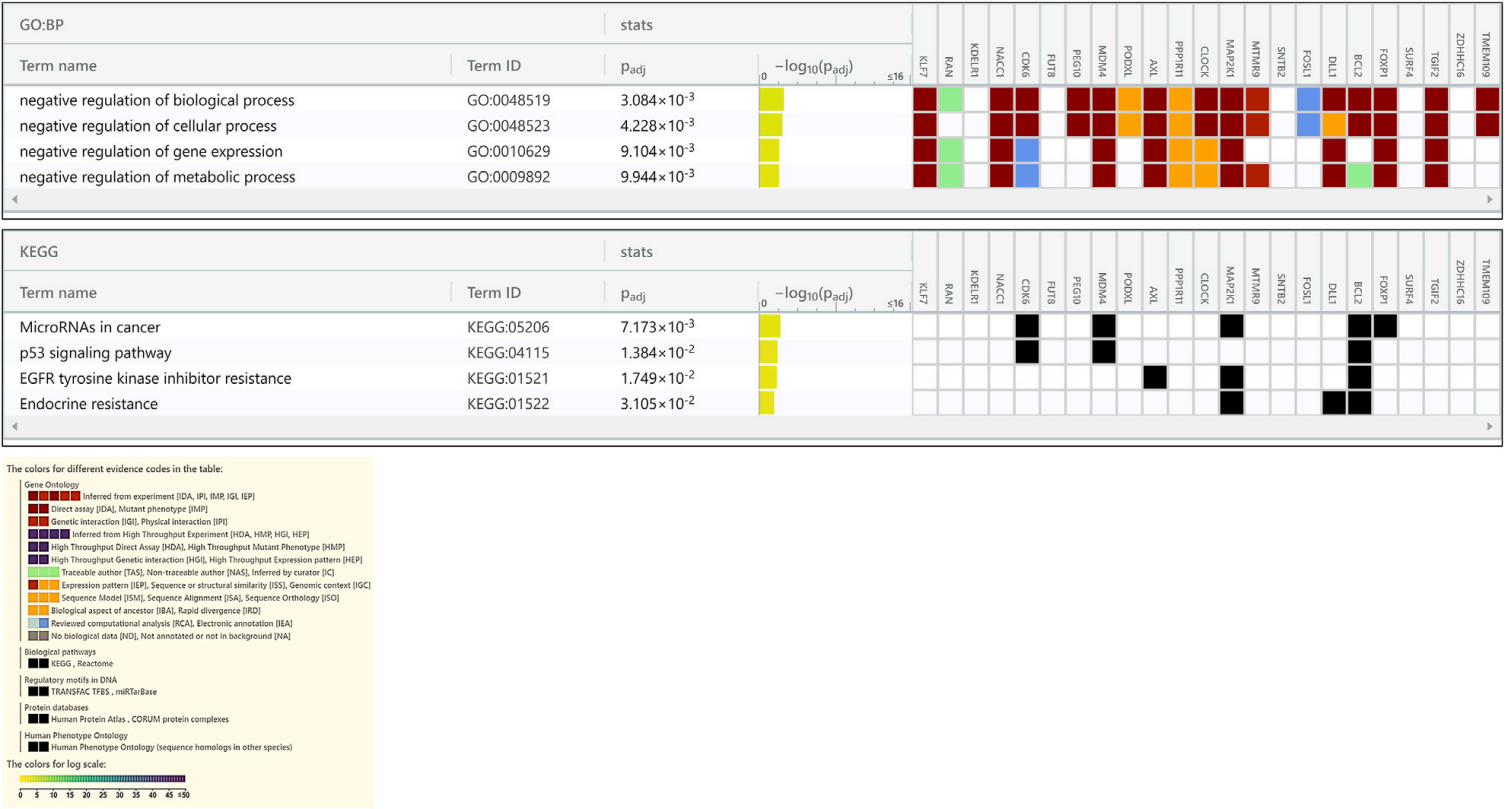
Enrichment in GO biological processes and KEGG pathways of target genes of miRNAs down regulated in PE. The GO biological processes and KEGG pathways were performed using g:Profiler for target genes of miRNAs down regulated in PE, with an adjusted *p* < 0.05. For each category, the number of genes is indicated by the length of horizontal bars.

Enrichment analyses showed that the 24 gene targets from the 3 up regulated miRNAs in PE were involved in multiple biological processes and reaction pathways (Figure 4A and Supplementary Figure S3). Biological processes associated with negative regulation of cellular processes (Figure 4B), and pathways related to MAPK, NOTCH, anti-apoptosis, and organelle trafficking (Figure 4C) were enriched. Similarly, the 18 predicted gene targets from the 3 downregulated miRNAs in PE were associated with biological processes and signaling pathways (Figures 5A, 6). There was overwhelming enrichment of biological processes (Figures 5B, 6) and pathways (Figures 5C, 6) related to cell cycle progression, proliferation, and WNT signaling. Cumulatively, the enrichment analyses highlighted the possibility that the differentially expressed up and down regulated miRNAs in PE or H/R are impacting signaling pathways related to cell growth, proliferation, survival, and cytokine secretion, which might be contributing to the observed pathophysiological changes *in vitro* and *in vivo*.

### Modulating Expression of miR-449a Can Attenuate Protective Effect of Exogenous uPA in the Hypoxia/Reoxygenation Model *in vitro*

**FIGURE 7:**
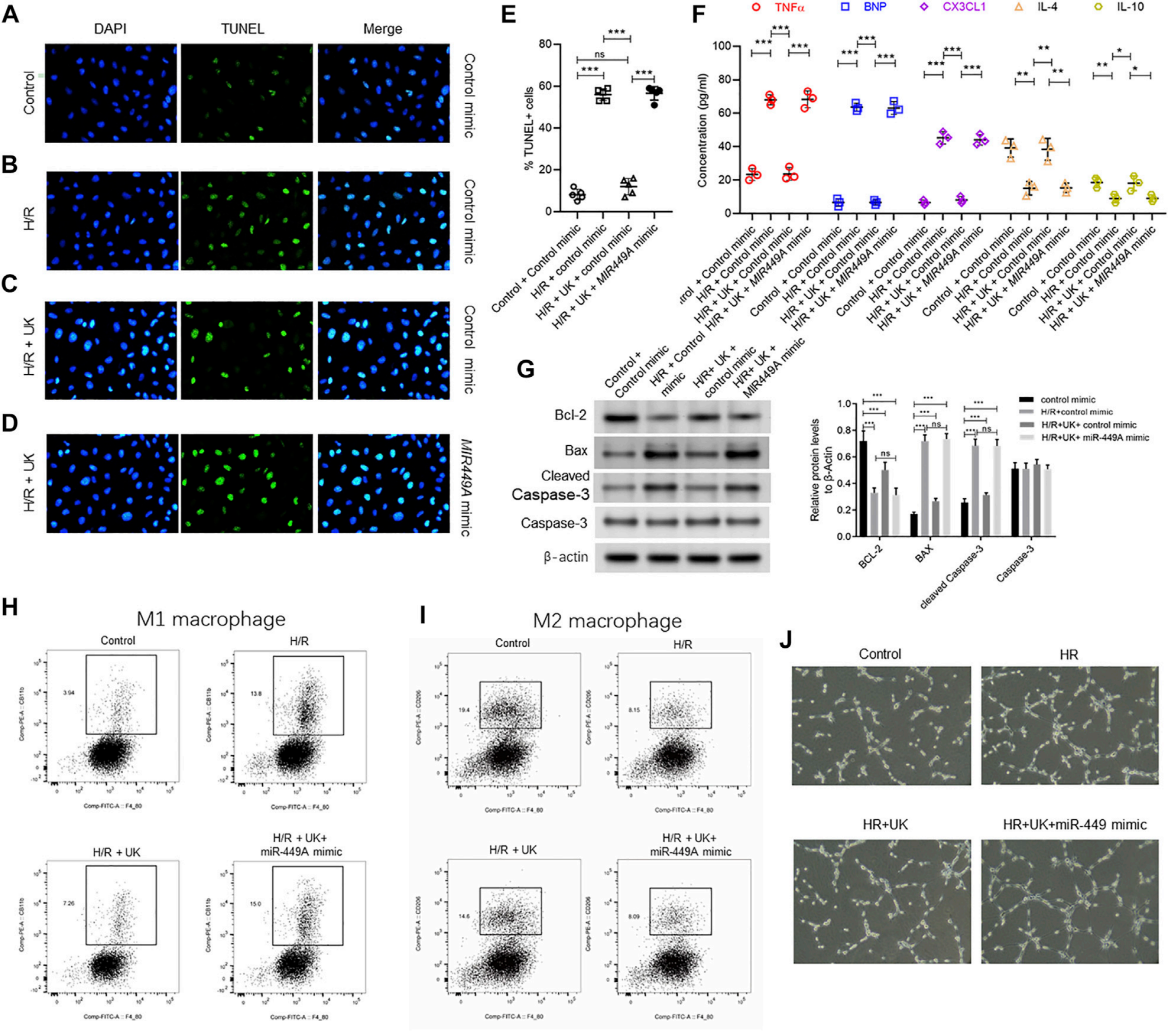
Ectopic expression of miR-449a inhibited protective effect of uPA on hypoxia/reoxygenation (H/R) model *in vitro*. After transfection with either a control or miR449a mimic, the APE model in BEAS-2B cells *in vitro* were subjected to hypoxic conditions for 12 h followed by reoxygenation for 12 h 72 h **(A–D)** Urokinase inhibits H/R induced cell death, and overexpression of miR-449a inhibited the role of urokinase. Representative images from TUNEL assay in control mimic-transfected BEAS-2B cells grown under control conditions **(A)** or subjected to H/R **(B)** or subjected to the combination of H/R and United Kingdom **(C)**, and miR449a mimic-transfected BEAS-2B cells subjected to H/R and treated with uPA **(D)**. **(E)** Quantification of images in ***A-D***. ****p < 0.0001, *ns*, not significant (*n* = 5). **(F)** Secretion of indicated cytokines in the cell supernatant was determined using Luminex assay. Pre-transfection with miR449a mimic inhibited urokinase-mediated decrease in cytokine secretion post-induction of H/R. ****p < 0.0001, *ns*, not significant (*n* = 3). **(G)** overexpression of miR-449a inhibited the effect of uPA on apoptosis in H/R model. Western blot images (left) and their quantified results (right) in BEAS-2B cells with different treatments. **(H–J)** Urokinase inhibits H/R induced changes of specific immune cells M1 macrophages **(H)** and M2 macrophages **(I)**, and overexpression of miR-449a inhibited the role of urokinase by FACS analysis. **(J)** Representative images of specific immune cells under different conditions.

MiR-449a was chosen to verify the hypothesis that miRNAs expression could attenuate protective effect of exogenous uPA in the H/R model *in vitro*, as miR-449a had the largest number of predicted target genes among the 3 downregulated miRNAs (Supplementary Figure S2). BEAS-2B cells were transfected with control mimic or miR449A mimic 72 h before H/R induction. Compared to control cells transfected with control mimic, H/R significantly induced cell death and increased expression of Bax and cleaved Caspase-3, but decreased bcl-2 expression (Figures 7A,B,E,G). The apoptosis and related protein expressions induced by H/R could be attenuated by uPA treatment (Figures 7A–C,E,G). However, treatment with uPA failed to inhibit cell death after H/R induction in BEAS-2B cells pre-transfected with miR449A mimic (Figures 7C–E,G). Similarly, uPA attenuated the levels of inflammatory cytokines and chemokine TNF-α, BNP, CX3CL1, IL-4, and IL-10, as well as specific immune cells M1 macrophages and M2 macrophages in cell supernatant of BEAS-2B cells subjected to H/R, but miR449A mimic transfection prevented decrease in these inflammatory cytokines, chemokine, and immune cells (Figures 7F,H–J). These results provide evidence that protective effect of exogenous uPA during H/R or APE is related to the expression of miR-449a.

## Discussion

Radiological imaging inclusive of venous ultrasonography, CT angiography, pulmonary venous angiography, and biochemical determination of serum D2D levels are clinically used to diagnose APE (Goldhaber and Elliott, 2003; Kearon, 1998; Xiao et al., 2011). Although with high specificity, D2D determination lacks specificity to assess APE (Goldhaber and Elliott, 2003; Kearon, 1998), hence additional diagnostic and prognostic biomarkers, which can be used alone or in combination with D2D are necessary to provide higher specificity. Indeed, miR-134 and miR-1233, either alone or in combination, have been shown to provide better diagnostic potential of COPD-associated APE (Xiao et al., 2011; Kessler et al., 2016; Peng et al., 2020). However, there are as yet no studies to explore differential miRNA expression before and after uPA treatment in APE, even though uPA has been a choice for APE treatment. Our analysis reveals that miR-34a-5p, miR-324-5p, miR-331-3p are up regulated following H/R or APE induction *in vitro* and *in vivo*, respectively, whereas miR-429, miR-491-5p, and miR-449a are down regulated under the same conditions.

More importantly, the expression of the mentioned 6 miRNAs were restored to levels comparable to control conditions following treatment with uPA, indicating their potential role in mediating the protective effect of uPA in PE. It is imperative to validate the roles of these 6 miRNAs in additional *in vivo* models of PE and ultimately in longitudinal patient samples with and without uPA treatment. Furthermore, whether the same set of miRNAs regulates effect of uPA on other cases of uPA/uPAR mediated pathophysiology, like vascular remodeling, cardiovascular disorders, and cancer, remains to be determined.

The processes and pathways related to cell cycle progression, cell growth, proliferation, and apoptosis were enriched, and each of them would be associated with pathophysiological changes observed in APE pulmonary tissue. Furthermore, merely overexpressing miR-449a was observed to mitigate the *in vitro* protective effect of uPA in BEAS-2B cells after H/R induction. It needs to be determined whether overexpression of the other 2 down regulated miRNAs, miR-429, and miR-491-5p, have a similar effect or synergistic effect. In addition, the effect of the knockdown of the upregulated miRNAs, miR-34a-5p, miR-324-5p, and miR-331-3p, on APE after uPA treatment also needs to be further studied.

One limitation of our study is that an unbiased miRNA profiling hasn’t been performed. The rationale for screening hypoxia specific miRNA array is that major pathophysiological changes of PE are associated with emboli-mediated hypoxia in bronchial tissue (Goldhaber and Elliott, 2003; Kearon, 1998). *CDK6* was predicted to be a target of both the up regulated miR-34a-5p and the down regulated miR-449a, indicating regulation of cell cycle is central to APE and uPA treatment potentially works *via* cell cycle regulation. There was significant enrichment of pathways related to organelle trafficking and endocytosis in our study. It has been shown that uPA interacts with its cognate receptor uPAR at the cell surface, inducing nuclear translocation of uPAR and subsequent downstream activation of signaling pathways vascular remodeling (Kiyan et al., 2009; Kiyan et al., 2012). Therefore, it needs to be determined if similar mechanisms are operative in APE. Moreover, the role of overexpressions of miR-449a in APE-induced mice with or without u-PA treatment, hasn’t been studied this time, which needed further experiment to explore the role of miR-449a *in vivo*.

In conclusion, our study reveals 6 differential expression miRNAs during PE induction *in vitro* and *in vivo*. These miRNAs are predicted to target genes associated with critical functions in cell cycle progression. Treatment with uPA reverses the expression of these 6 miRNAs to levels comparable to control conditions, indicating that effect of uPA might be affected by these miRNAs. Indeed, ectopic overexpression of miR-449a, one of the downregulated miRNAs, was sufficient to inhibit uPA-mediated protective effect against H/R-mediated changes *in vitro*. These miRNAs thus might be utilized as biomarkers in PE, especially in the treatment with uPA.

## Data Availability

The original contributions presented in the study are included in the article/Supplementary Material, further inquiries can be directed to the corresponding author.
