# Supplementary figures and images for: MicroRNA 449a can Attenuate Protective Effect of Urokinase Against Pulmonary Embolism
Source: Front Pharmacol. 2022 Apr 28;13:713848. doi: 10.3389/fphar.2022.713848 (PMC9095938; doi:10.3389/fphar.2022.713848)

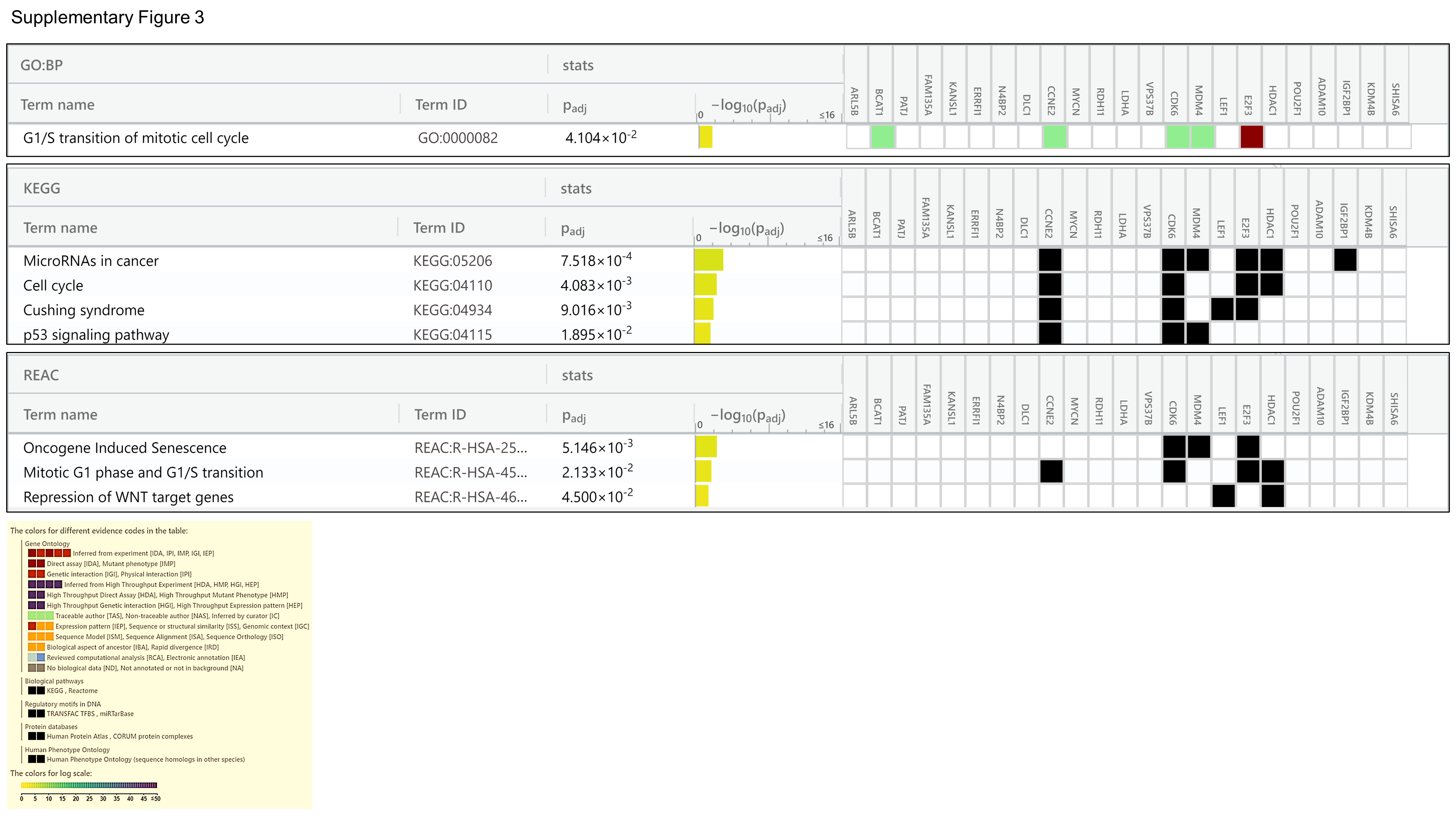

Supplement: Supplementary file 2 [file Image3.TIF]

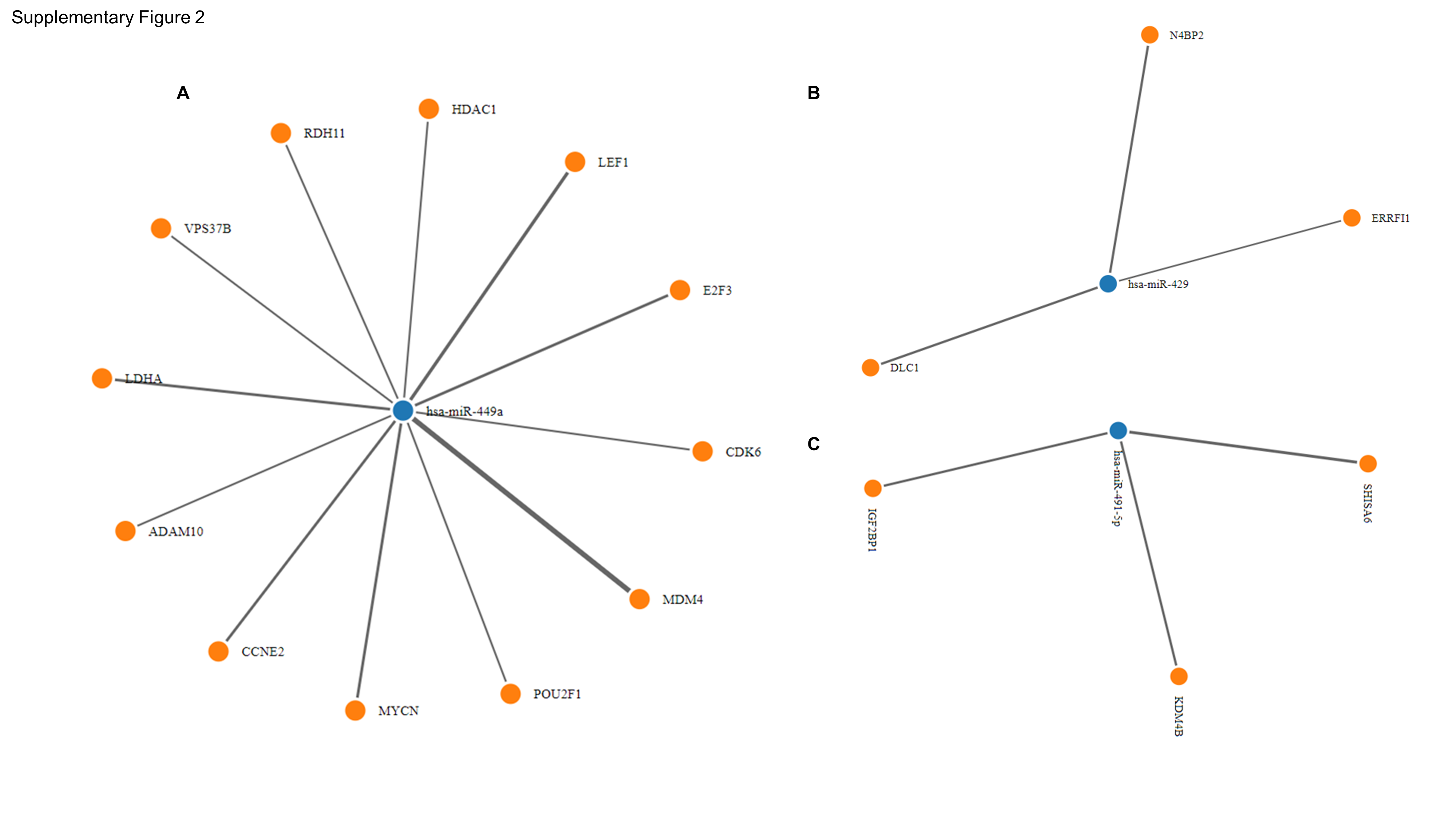

Supplement: Supplementary file 3 [file Image2.TIF]

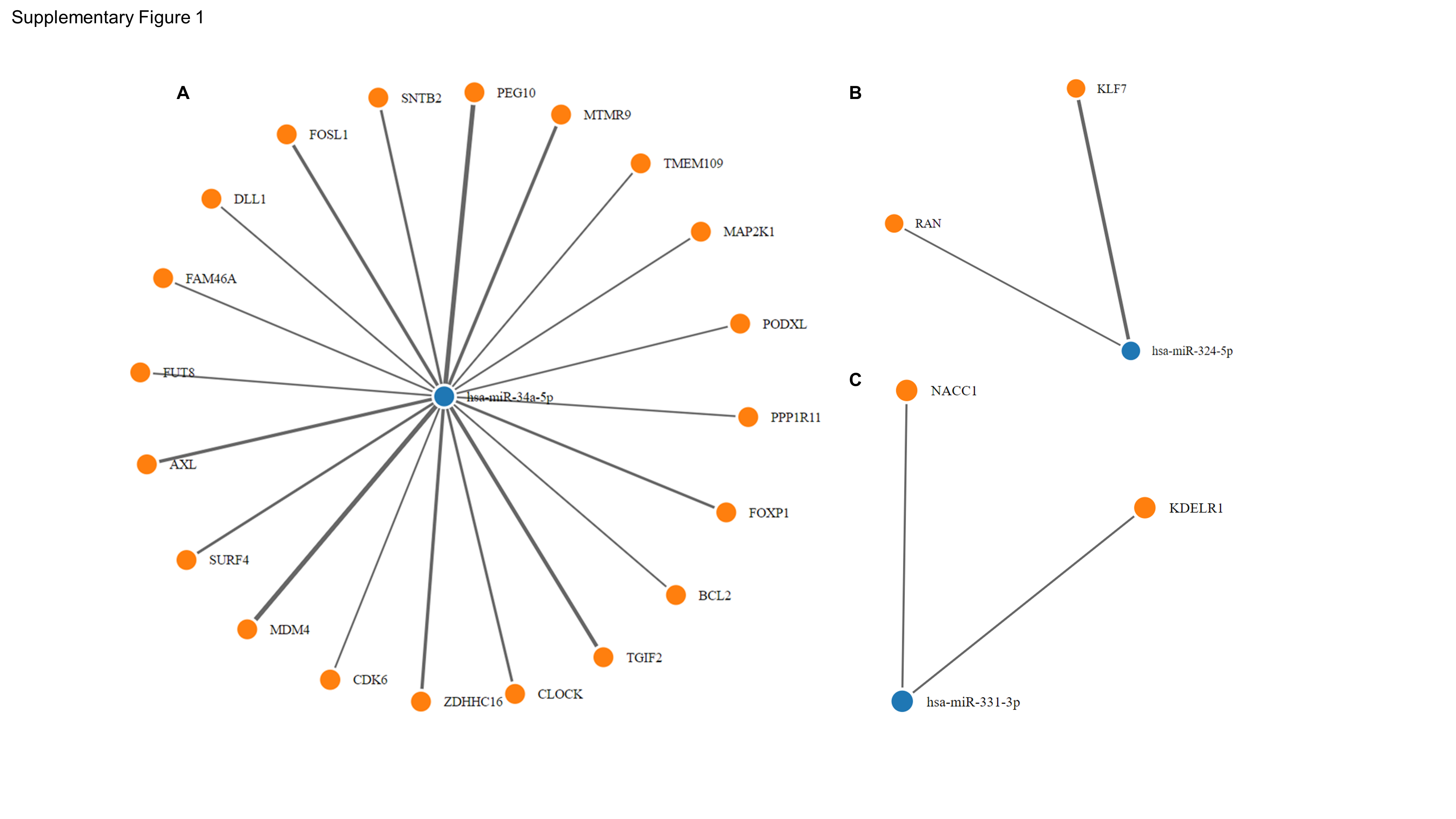

Supplement: Supplementary file 4 [file Image1.TIF]
